# Supplementary material for: The Actin-Family Protein Arp4 Is a Novel Suppressor for the Formation and Functions of Nuclear F-Actin
Source: Cells. 2020 Mar 19;9(3):758. doi: 10.3390/cells9030758 (PMC7140684; doi:10.3390/cells9030758)
Supplement: Supplementary file 1 [file cells-09-00758-s001.pdf]

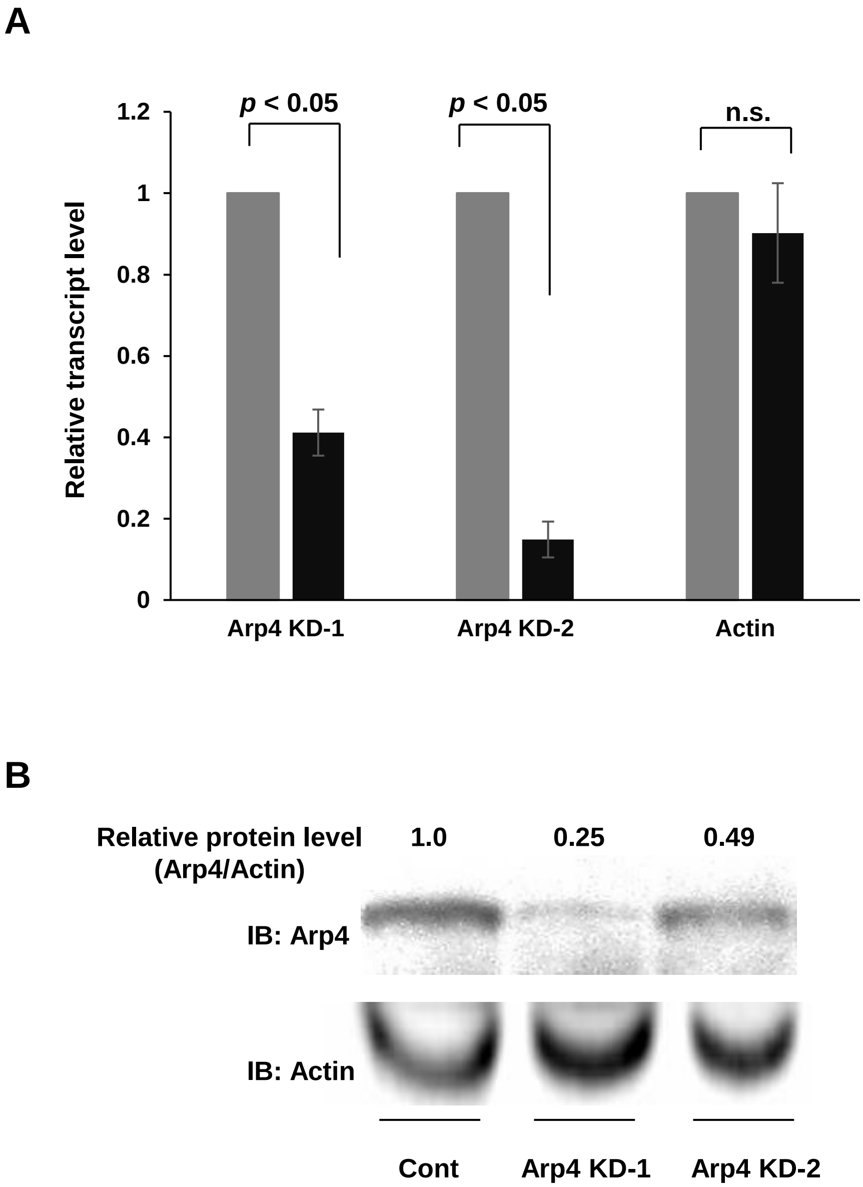

Figure S1: (A) The amount of Arp4 and actin mRNAs in the indicated Arp4-knochdown cells was determined by quantitative RT-PCR. Two independent siRNAs (siArp4-1 and siArp4-2) were used for Arp4 knockdown. Data shown are mean ± SEM; n = 3. (B) Total cellular proteins, extracted from the indicated cells, were analyzed by Western blot to determine the amount of Arp4 and actin. Control, cells expressing control siRNA; Arp4 KD-1, cells expressing Arp4-1 siRNA; Arp4 KD-2, cells expressing Arp4-2 siRNA.

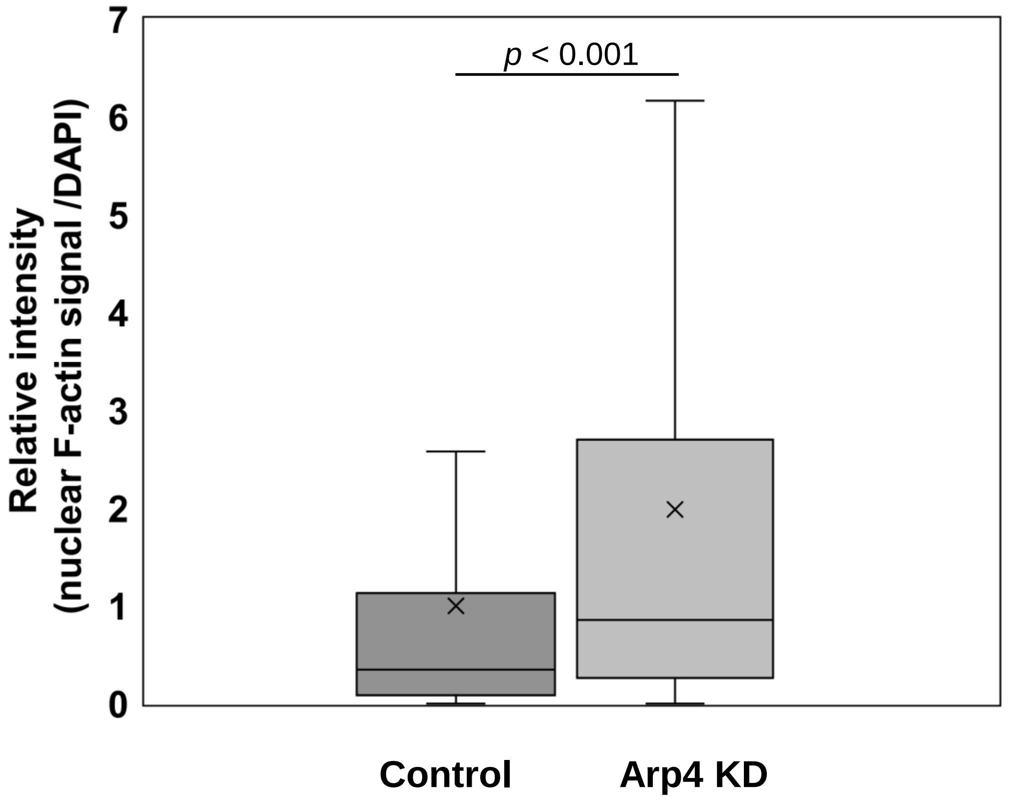

Figure S2: Nuclear F-actin was detected in NIH3T3 cells transfected with control siRNA (Control) or Arp4-1 siRNA (Arp4 KD), as in Fig. 1A. Nuclear F-actin formation was detected by expressing the nuclear actin probe nAC-mCherry. The intensity for nuclear F-actin, which is higher than that of nuclear background, was measured, the relative fluorescence intensity of nuclear F-actin to that of DAPI was determined using Fiji image analysis software. The result is shown as a box plot. For the quantification, 236 control cells and 208 Arp4 KD cells were analyzed.

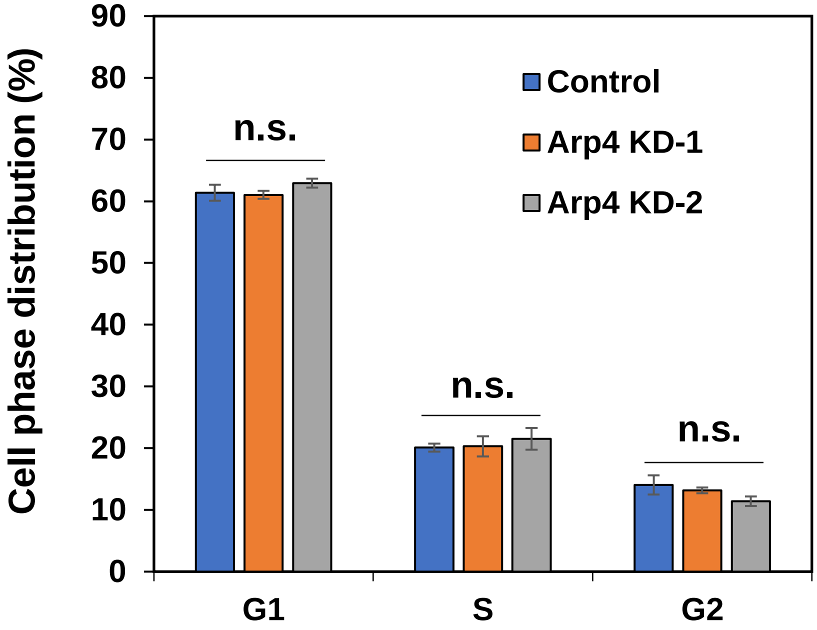

Figure S3: Cell cycle analysis of NIH3T3 cells transfected with control siRNA (Cont), Arp4-1 siRNA (Arp4 KD-1) or Arp4-2 siRNA (Arp4 KD-2). Cell cycle analysis was performed using a Propidium Iodide Flow Cytometry Kit (Abcam), following the manufacturer's recommendations. NIH3T3 cells were transfected with control siRNA, Arp4-1 siRNA or Arp4-2 siRNA by electroporator and cultured for 24 hr. The cells were fixed with 66% ethanol and treated with propidium iodide (PI) staining and RNase solution on ice for 30 . Data from 30,000 cells per sample were collected and analyzed on a BD Accuri C6 flow cytometer (BD). The percentage of the cells in cycle phases was determined using BD Accuri C6 software (BD). Data shown are mean ± SEM; n = 3.

Table S1: Primers used in this study.

GAPDH (human)-F	5-TGCACCACCAACTGCTTAGC-3
GAPDH (human)-R	5-GGCATGGACTGTGGTCATGAG-3
Actin (human)-F	5-ATCGTCCACCGCAAATGCTTCTA-3
Actin (human)-R	5-AGCCATGCCAATGTGATGTTCTT-3
Arp4 (human)-F	5-GAGTTCCCAAGCTTCTACCTTCCT-3
Arp4 (human)-R	5-CATTCTACAAAAGATGGTCATTCTTTTC-3
Exportin6 (human)-F	5-AACAATGCCCACACAGTGAA-3
Exportin6 (human)-R	5-AACTCCACCACAGGGAACTG-3
Importin9 (human)-F	5-ACTGGCAATCCGTCAGCTGGC-3
Importin9 (human)-R	5-GGCAATAGCTCCCGGATAACA-3
IGF-1 (human)-F	5-GACATGCCCAAGACCCAGAAGGA-3
IGF-1 (human)-R	5-CGGTGGCATGTCACTCTTCACTC-3
OCT-4 (human)-F	5-GACAACAATGAGAACCTTCAGGAGA-3
OCT-4 (human)-R	5-CTGGCGCCGGTTACAGAACCA-3
SOX2 (human)-F	5-ACATGTGAGGGCTGGACTGCGAAC-3
SOX2 (human)-R	5-GAAGCGCCTAACGTACCACTAGAAC-3
UTF1 (human)-F	5-AGCAGATCCGGAAGCTCATGGG-3
UTF1 (human)-R	5-TCCTCGGGGATGCAGGTG-3
GDF3 (human)-F	5-GCCATCAAAGAAAGGAACA-3
GDF3 (human)-R	5-TTGAGAGTCACCACCAGCAG-3
LEFTY1 (human)-F	5-CTGCCCATGATCGTCAGCATC-3
LEFTY1 (human)-R	5-AGACCACCTCTATGCACACGT-3
ACTA2 (human)-F	5-GTGCTGGACTCTGGAGATGG-3
ACTA2 (human)-R	5-AATAGCCACGCTCAGTCAGG-3
CCND1 (human)-F	5-CTGGAGGTCTGCGAGGAACA-3
CCND1 (human)-R	5-CCTTCATCTTAGAGGCCACGAA-3
Axin2 (human)-F	5-AGTGTGAGGTCCACGGAAAC-3
Axin2 (human)-R	5-CTTCACACTGCGATGCATTT-3
TCF-1(human)-F	5-TGACCTCTCTGGCTTCTACT-3
TCF-1(human)-R	5-TTGATGGTTGGCTTCTTGGC-3
